# *C9orf72* Hexanucleotide Expansions Are Associated with Altered Endoplasmic Reticulum Calcium Homeostasis and Stress Granule Formation in Induced Pluripotent Stem Cell‐Derived Neurons from Patients with Amyotrophic Lateral Sclerosis and Frontotemporal Dementia

**DOI:** 10.1002/stem.2388

**Published:** 2016-05-04

**Authors:** Ruxandra Dafinca, Jakub Scaber, Nida'a Ababneh, Tatjana Lalic, Gregory Weir, Helen Christian, Jane Vowles, Andrew G.L. Douglas, Alexandra Fletcher‐Jones, Cathy Browne, Mahito Nakanishi, Martin R. Turner, Richard Wade‐Martins, Sally A. Cowley, Kevin Talbot

**Affiliations:** ^1^ Nuffield Department of Clinical Neurosciences; ^2^ Department of Physiology, Anatomy and Genetics; ^3^ James Martin Stem Cell Facility, Sir William Dunn School of Pathology, University of OxfordOxfordUnited Kingdom; ^4^ Research Center for Stem Cell Engineering, National Institute of Advanced Industrial Science and Technology (AIST)TsukubaIbarakiJapan

**Keywords:** Amyotrophic lateral sclerosis, Frontotemporal dementia, Induced pluripotent stem cells, *C9orf72*, Calcium dysregulation, Motor neurons

## Abstract

An expanded hexanucleotide repeat in a noncoding region of the *C9orf72* gene is a major cause of amyotrophic lateral sclerosis (ALS), accounting for up to 40% of familial cases and 7% of sporadic ALS in European populations. We have generated induced pluripotent stem cells (iPSCs) from fibroblasts of patients carrying *C9orf72* hexanucleotide expansions, differentiated these to functional motor and cortical neurons, and performed an extensive phenotypic characterization. In *C9orf72* iPSC‐derived motor neurons, decreased cell survival is correlated with dysfunction in Ca^2+^ homeostasis, reduced levels of the antiapoptotic protein Bcl‐2, increased endoplasmic reticulum (ER) stress, and reduced mitochondrial membrane potential. Furthermore, *C9orf72* motor neurons, and also cortical neurons, show evidence of abnormal protein aggregation and stress granule formation. This study is an extensive characterization of iPSC‐derived motor neurons as cellular models of ALS carrying *C9orf72* hexanucleotide repeats, which describes a novel pathogenic link between *C9orf72* mutations, dysregulation of calcium signaling, and altered proteostasis and provides a potential pharmacological target for the treatment of ALS and the related neurodegenerative disease frontotemporal dementia. Stem Cells *2016;34:2063–2078*

**Significance Statement**

Alterations in the *C9orf72* gene have been identified as the most common underlying genetic abnormality in frontotemporal dementia (FTD) and amyotrophic lateral sclerosis (ALS) patients, accounting for approximately 40% of familial cases and providing a clear link between the two conditions. This study represents an extensive characterization of the cellular processes affected by alterations in *C9orf72* using iPS‐derived motor neurons and cortical neurons from ALS/FTD patients. Our study revealed that the *C9orf72* mutation induces disease‐specific alterations in intracellular calcium dynamics, changes in morphology of essential cellular compartments, along with high levels of protein aggregates in both affected cell types. Our observations represent the first direct comparison between iPS‐derived motor neurons and cortical neurons of *C9orf72* cases, and they provide the foundation for further studies of the mechanism of the disease causing mutation and for the exploration of disease‐modifying therapies.

## Introduction

A hexanucleotide (GGGGCC) expansion in the first intron of the *C9orf72* gene accounts for approximately 40% of cases of familial amyotrophic lateral sclerosis (ALS), up to 7% of sporadic ALS, and approximately 20% of familial frontotemporal lobar degeneration, establishing a firm genetic link between ALS and frontotemporal dementia (FTD) 1, 2, 3. The expansion is located in an intronic or promoter region upstream of the *C9orf72* coding sequence, and the number of (GGGGCC)_*n*_ hexanucleotide repeats ranges between 100 and 4,000 repeats in patients 1, 2, 4. *C9orf72*‐associated cases exhibit distinctive neuropathological changes characterized by abundant cytoplasmic and intranuclear inclusions immunopositive for ubiquitin and sequestosome‐1/p62, suggesting disrupted protein degradation 5, 6, 7.

While the function of the *C9orf72* gene and the pathogenic mechanisms of the hexanucleotide expansion are currently unknown, several hypotheses have been proposed. A toxic gain of function mechanism, mediated by the accumulation of (GGGGCC)_*n*_‐rich RNA transcripts within nuclear foci observed in the frontal cortex and spinal cord of patients, might lead to sequestration of RNA binding proteins and disruption of the translation of diverse mRNAs 1 or increased nucleolar stress 8. Alternatively, reduced transcription of *C9orf72* could lead to neurodegeneration by interfering with the constitutive function of the protein 1, 9. Lastly, repeat‐associated non‐ATG (RAN) translation, occurring in the absence of an initiating ATG codon, over the GGGGCC repeat expansion has been shown to produce homopolymeric proteins prone to aggregation 10, 11.

The generation of human motor neurons (MNs) in culture from induced pluripotent stem cells (iPSC), reprogrammed from skin fibroblasts of patients with neurodegenerative diseases such as ALS, offers a potentially powerful tool with which to study the key pathological processes in MN degeneration and for screening drugs of potential therapeutic benefit. Previous studies have demonstrated that RNA foci and RAN‐translation products can be detected in iPSC‐derived MNs from ALS/FTD patients with *C9orf72* hexanucleotide expansions 12, 13, 14. Evidence of defects in autophagy, sequestration of RNA‐binding proteins by the expanded repeat, changes in gene transcription, and altered neuronal excitability suggest that these models can display disease‐relevant phenotypes which can be corrected by targeting the expanded RNA with antisense oligonucleotides 13, 14.

Calcium (Ca^2+^) dysregulation is believed to play an important role in the pathophysiology of ALS 15, and Ca^2+^ overload in the cytoplasm of neurons is a potential mechanism that may link excitotoxicity to neuronal death 16. The endoplasmic reticulum (ER) is the largest intracellular Ca^2+^ store and high ER Ca^2+^ concentration plays an essential role in the activity of protein synthesis and processing. Disturbances in ER Ca^2+^ homeostasis have been linked to chronic activation of the ER stress response and downstream compensatory mechanisms to protect the cell, such as the unfolded protein response (UPR) and autophagy 17. ER‐stress induced cell death can proceed in both Ca^2+^‐dependent and Ca^2+^‐independent ways. The antiapoptotic proteins Bcl‐2 and Bcl‐XL are essential modulators of Ca^2+^ signaling in the cell, mediating the decrease and release of Ca^2+^ in the ER thereby protecting mitochondria from Ca^2+^ overload 18, 19.

In order to explore whether altered Ca^2+^ homeostasis and ER stress are cellular phenotypes associated with *C9orf72*‐related ALS/FTD, we generated iPSC lines from skin fibroblasts from patients carrying *C9orf72* hexanucleotide expansions and differentiated them to MNs and cortical neurons (CNs). Here we show that in *C9orf72* iPSC‐derived MNs loss of Ca^2+^ homeostasis is associated with a decrease in mitochondrial potential and increase in ER stress. Increased susceptibility to cell death was accompanied by reduced levels of the antiapoptotic Bcl‐2 and Bcl‐X_L_ proteins and upregulation of the proapoptotic protein BAK. Furthermore, both *C9orf72* MNs and CNs showed elevated levels of SQST‐1/p62 and the formation of poly‐A‐binding protein (PABP) positive stress granules. Our results indicate disease‐specific alterations in MNs and CNs of ALS/FTD patients carrying *C9orf72* repeat expansions and reveal a novel pathogenic link between *C9orf72*, Ca^2+^ dysregulation, ER stress, and loss of proteostasis.

## Materials and Methods

### Ethics Statement

The human iPSC lines used for this study were derived from human skin biopsy fibroblasts, following signed informed consent, with approval from the South East Wales Research Ethics Committee (REC ref no. 12/WA/0186) and the South Central Berkshire Research Ethics Committee, U.K. (REC 10/H0505/71).

### Generation and Culture of iPSC Lines

In this study, we used six iPSC‐derived lines from four healthy controls: NHDF‐1; OX3‐9; OX1 clones 19 and 61; AH017 (clones 3 and 13) and six lines from 3 *C9orf72* patients: C9‐T2 (clones 6 and 7); C9‐7245 (clones 1 and 3); C9‐02 (clones 2 and 10). All iPSC lines were derived from skin biopsy fibroblasts in the James Martin Stem Cell Facility, University of Oxford, under standardized protocols to minimize any potential variation attributable to laboratory differences and/or handling. Fibroblasts tested negative for mycoplasma using MycoAlert (Lonza, United kingdom, www.lonza.com/) and the derived iPSC lines also tested negative using the same test. The control iPSC line iPS‐NHDF‐1 [44 year‐old female] 20 and iPS‐OX1‐19 [36M] 21, derived with Yamanaka retroviruses, have been published previously. C9‐T2‐6, C9‐T2‐7 [62M], 7245‐1, 7245‐3 [48F] and the control lines iPS‐OX3‐9 [49M] and iPS AH017‐13 [67F] (previously published 22) were derived using the SeVdp(KOSM)302L Sendai virus system 23, 24. This contains the genes for all four reprogramming transcription factors (Klf4, Oct4, Sox2, c‐Myc) expressed from a single transcript, packaged into a single Sendai virus, ensuring consistency of gene dosage ratio. Sendai virus is a negative strand RNA virus that is retained in the cytoplasm. As such, the likelihood of insertion of genetic material into the genome is negligible. This version of the system also contains a target for mir302; mir302 is expressed in pluripotent cells, but not in the originating fibroblasts, so the viral RNA will be detected and degraded once the cells have reprogrammed to pluripotency, ensuring complete removal of exogenous genetic material within a few passages. AH017‐13 [67F], OX1‐61 [36M], C902‐02, C902‐10 [62F] were derived using the commercially available Sendai virus‐based reprogramming system Cytotune, which contains the reprogramming genes Klf4, Oct4, Sox2, c‐Myc packaged into individual viruses (Life Technologies, Rockville, MD, http://www.lifetech.com, used according to the manufacturer's instructions).

Transduced fibroblasts were transferred onto mitotically inactivated mouse embryonic feeder cells (MEFs; derived in‐house from outbred Swiss mice established and maintained at the Department of Pathology, Oxford 25, 26, or MEFs purchased from Merck, Whitehouse Station, NY, http://www.merck.com, derived from CF1 mice) on 0.1% gelatin coated plates on day 2 and were cultured in iPS medium consisting of knock‐out Dulbecco's modified Eagle's medium (DMEM) (Invitrogen, Carlsbad, CA, http://www.invitrogen.com), 10% knock‐out‐serum replacement (Invitrogen), 2 mM Glutamax‐I (Gibco, Grand Island, NY, http://www.invitrogen.com), 100 U/mL penicillin (Invitrogen), 100 µg/mL streptomycin (Invitrogen), 1% nonessential amino acids (Invitrogen), 0.5 mM β‐mercaptoethanol (Invitrogen), 10 ng/mL basic fibroblast growth factor (bFGF) (R&D, Minneapolis, http://www.rndsystems.com). Medium was replaced (50%) daily, and substituted with MEF‐conditioned medium from day 10 onward. Colonies displaying iPSC morphology were picked on day 21–28 and transferred onto MEFs by manual dissection every 5–7 days. iPSC lines were adapted to feeder‐free conditions onto Matrigel‐coated plates (BD Matrigel hESC‐qualified Matrix) in mTeSR1 (100% daily medium change, StemCell Technologies, Vancouver, BC, Canada, http://www.stemcell.com). Bulk and routine passaging was by 0.5 mM EDTA 27 to make large‐scale, quality‐controlled, cryopreserved master‐stocks (between p10 and 20). The initial master‐stock underwent all the QC tests described, and all subsequent submaster‐stocks underwent single nucleotide polymorphism (SNP) analysis for tracking and genome integrity. The number of feeder‐free passages was kept to a minimum, to ensure that differentiation experiments were set up within a narrow window of passage numbers. If single‐cell dissociation was required for particular applications, medium was supplemented with Rock inhibitor Y27632 (10 µM; Calbiochem, San Diego, CA, http://www.emdbiosciences.com) on the day of passage.

### Assessment of Genome Integrity and Tracking

Genome integrity was assessed by Illumina Human CytoSNP‐12v2.1 beadchip array (300,000 markers) or Illumina HumanOmniExpress‐24 (700,000 markers) and analyzed using KaryoStudio and GenomeStudio software (Illumina). SNP deviations in the iPSC lines were compared to the original pool of fibroblasts. This also confirmed the identity of the iPSC to the original fibroblasts. SNP datasets have been deposited in Gene Expression Omnibus (GEO) under the accession number GSE64582.

### Sendai Clearance Assay

Clearance of Sendai virus from reprogrammed cell lines was confirmed by polymerase chain reaction (PCR). RNA was extracted using an RNeasy kit (Qiagen, Hilden, Germany, http://www1.qiagen.com) from iPSC, from fibroblasts (negative control), and from fibroblasts infected with Sendai reprogramming viruses 5 days previously (positive control). Reverse transcription was carried out using a RetroScript kit (Ambion, Austin, TX, http://www.ambion.com), using 2 µg template RNA in 20 µL reaction volume. 2 µL of 1:10 dilution of cDNA product was used in a 25 µL PCR reaction. For lines reprogrammed with SeVdp(KOSM)302L, quantitative reverse transcriptase PCR (qRT‐PCR) was carried out on an Applied Biosystems StepOne Plus Real Time PCR machine, with StepOne software, using Applied Biosystems 2×SYBR green PCR mix+ROX and 60 degree Celsius anneal. Target gene transcript levels were compared to actin β control (actin β primers, Eurogentec) and subsequently to the positive control 28. Sendai‐specific primers were 5′‐AGACCCTAAGAGGACGAAGACAGA‐3′ and 5′‐ACTCCCATGGCGTAACTCCATAG‐3′. For lines reprogrammed with the Cytotune Sendai reprogramming system, PCR was carried out according to the manufacturers' instructions, with a primer pair for each of the four reprogramming viruses and also for the backbone, using actin β as a positive control for each line (actin β primers, Eurogentec), and products were visualized by running on a 1.5% agarose gel with ethidium bromide, and 2‐log ladder or 100 bp ladder (New England Biolabs). Primer pairs were: SeV F: GGA TCA CTA GGT GAT ATC GAG C; R: ACC AGA CAA GAG TTT AAG AGA TAT GTA TC; SOX2 F: ATG CAC CGC TAC GAC GTG AGC GC; R: AAT GTA TCG AAG GTG CTC AA; KLF4 F: TTC CTG CAT GCC AGA GGA GCC C; R: AAT GTA TCG AAG GTG CTC AA; c‐MYC F: TAA CTG ACT AGC AGG CTT GTC G; R: TCC ACA TAC AGT CCT GGA TGA TGA TG; OCT4F: CCC GAA AGA GAA AGC GAA CCA G; R: AAT GTA TCG AAG GTG CTC AA.

### PluriTest

RNA was extracted from iPSC using an RNeasy kit (Qiagen) for Illumina HT12v4 transcriptome array analysis. The data files were then uploaded to www.pluritest.org and scored for pluripotency, as previously described. The Illumina HT12v4 transcriptome array results have been deposited in the GEO under accession number GSE64583.

### Flow Cytometry

iPSC lines were assessed for expression of pluripotency markers by flow cytometry. Cells were lifted with TrypLE (Life Technologies), fixed with 4% paraformaldehyde (PFA) in phosphate‐buffered saline (PBS) for 10 minutes then permeabilized in methanol at −20°C. 0.5–1 × 10^6^ cells were then washed and stained in flow cytometry buffer consisting of PBS, human IgG (10 µg/mL Sigma), fetal calf serum (FCS) (1% Hyclone, Logan, UT, http://www.hyclone.com), and sodium azide (0.01%), with an antibody or an isotype‐matched control (with same fluorophore, from the same manufacturer, at 1 µg antibody per million cells) for 30 minutes. After the primary staining, cells were washed three times, and fluorescence was measured using a FACS Calibur (Becton Dickinson, Franklin Lakes, NJ, http://www.bd.com)—data from 10,000 cells were collected and analyzed using FlowJo software, gated on forward scatter‐side scatter (FSC‐SSC) dot plots, and plotted as histograms (*y* axis normalized to mode). The following antibodies (clone, isotype control, supplier) were used: TRA‐1‐60 (B119983, IgM‐488, Biolegend), NANOG (D73G4, IgG‐647, Cell Signaling Technology, Beverly, MA, http://www.cellsignal.com).

### Differentiation of iPSCs to MNs

MNs were differentiated from iPSCs using three different previously published protocols, with several modifications 29, 30. In one of the differentiation protocols, the differentiation process was initiated by neural induction of embryoid bodies (EBs) and was followed by neural progenitor stages and neural maturation. After 4 days in neural induction medium, the EBs were plated onto Geltrex‐coated plates, and caudalization of the neural progenitors was obtained by addition of retinoic acid (RA) to the neural differentiation medium at a concentration of 0.1 μM. After 10 days, the colonies displaying neural rosettes structures were isolated and expanded in suspension in the form of neurospheres in the presence of the ventralizing growth factor, sonic hedgehog at a concentration of 100 ng/mL. The protocol was modified at this step to enhance cell survival by addition of Rock inhibitor Y27632, which prevents cell detachment and is a widely used supplement in stem cell cultures 31, 32. After 2 weeks of culture as neurospheres, the cells were plated on Geltrex‐coated coverslips, and terminal differentiation was induced by addition of brain derived neurotrophic factor (BDNF 10 ng/mL), glial‐derived neurotrophic factor (GDNF, 10 ng/mL), insulin‐like growth factor (IGF‐1, 10 ng/mL), cyclic AMP (cAMP), and ascorbic acid 0.5μM, Sigma ascorbic acid. The differentiating MNs were cultured for another 3–4 weeks before functional assays were performed.

Using the second differentiation protocol 30, the iPSCs were cultured as monolayers, and neural induction was started by addition of compound c (1 μM) Tocris Bioscience, Bristol UK, https://www.tocris.com/ in DMEM/F12 and Neurobasal medium, supplemented with N2, B27, Heparin 0.2%. After 3 days, the medium was supplemented with RA (1 µM) and after another 3 days, smoothened agoinst (SAG) and purmorphamine (1 µM) were added and compound c removed. After 9 days in culture, the neural precursors were replated at a low density for differentiation and the medium was supplemented with the growth factors BDNF, GDNF and IGF‐1 (10 ng/mL), ascorbic acid and cAMP. After 20 days, the MNs were replated for experiments and allowed to mature for another 3–4 weeks before functional experiments.

MNs were differentiated using a third differentiation protocol 33, with extensive modifications. Monolayers of iPSCs were induced in DMEM/F12/Neurobasal with N2 supplement, B27 supplement, ascorbic acid (0.5 µM), 2‐mercaptoethanol (1×), compound C (3 µM). and Chir99021 (3 µM) for 4 days. After 4 days in culture, RA (1 µM) and SAG (500 nM) were added to the medium. The next day, Chir99021 and compound C were removed from the medium, and the cells were cultured for another 4–5 days. Neural precursors were then dissociated and plated at a low density for 7 days. The medium was supplemented with growth factors BDNF (10 ng/mL), GDNF (10 ng/mL), N‐[N‐(3,5‐Difluorophenacetyl)‐L‐alanyl]‐S‐phenylglycine t‐butyl ester (DAPT) (10 µM), and laminin (0.5 µg/mL). After 7 days, DAPT and laminin were removed from the medium, and the neurons were allowed to mature for another 2–4 weeks before functional experiments.

### Differentiation of iPSCs to CNs

The differentiation of iPSCs to CNs was performed following a previously established protocol 34. Briefly, the iPSCs were grown on Matrigel as monolayers, and neural differentiation was induced by changing the medium to basic neural medium DMEM/F12: Neurobasal 1:1, 1× N2, 1× B27, insulin 5 µg/mL (Sigma), nonessential amino acids 100 µM (Life Technologies), 2‐mercaptoethanol 100 µM, antibiotic‐antimycotic (Life Technologies) and supplementing it with 500 ng/mL Noggin (PeproTech, Rocky Hill, NJ, http://www.peprotech.com), and 10 µM SB431542 (Enzo Life Sciences). Medium was changed every 24 hours until neural rosettes could be observed (12–16 days). The neuroepithelial layer was lifted using Dispase (Life Technologies), and the medium was supplemented with 20 ng/mL Fibroblast growth factor 2 (FGF2) (PeproTech) for 4–6 days. The neural precursors were split using Accutase (Life Technologies) at a low density. The medium was then supplemented with 10 ng/mL BDNF and 10 ng/mL GDNF, while FGF2 was withdrawn. The neurons were allowed to extend processes and to mature for 60–70 days before functional assays.

### Immunocytochemistry

Mature MNs and CNs were fixed with 4% PFA‐PBS for 15 minutes and incubated with 10% goat serum for blocking for 1 hour at room temperature. The cells were incubated overnight at 4°C with rabbit anti‐cleaved‐caspase‐3 (Cell Signaling, 1:350), rabbit anti‐PABP (1:500), mouse anti‐Islet 1 (DSHB, Iowa City, IA, http://www.uiowa.edu/~dshbwww, 1:200), mouse or rabbit anti‐Tuj1 (Covance, Princeton, NJ, http://www.covance.com, 1:1,000), mouse anti‐p62 (Abcam, Cambridge, U.K., http://www.abcam.com, 1:500), rabbit anti‐GA (ProteinTech, 1:500), rabbit anti‐GP (ProteinTech, 1:500), rabbit anti‐PR (ProteinTech, 1:500), rabbit anti‐GR (ProteinTech, 1:500), goat anti‐TIA‐1 (Santa Cruz Biotechnology, Santa Cruz, CA, http://www.scbt.com, 1:500). After washing with 0.1% Triton‐X/PBS three times for 10 minutes, the samples were incubated with Alexa Fluor 488 or Alexa Fluor 568 conjugated goat anti‐rabbit or goat anti‐mouse secondary antibodies (Life Technologies) for 1 hour at room temperature. Nuclei were stained with 4',6‐Diamidino‐2‐Phenylindole (DAPI). Fluorescence was visualized using a confocal microscope Zeiss LSM.

### Immunoblotting

Cells were washed with PBS and then scraped in PBS. Cells were lysed in RIPA buffer (0.5% sodium deoxycholate, 0.1% SDS, 1% Nonidet P40 and 1% protease inhibitor cocktail (Sigma)), sonicated, and incubated on ice for 30 minutes. After centrifugation at 5,000*g* for 10 minutes, the supernatant was retained, and protein concentration was quantified using the BCA assay according to the manufacturer's instructions (Sigma). Protein was boiled in Laemmli buffer for 5 minutes at 95°C, and 10 μg of protein were loaded on gels. For Western blot analysis, lysates were resolved on a SDS‐PAGE (10% or 12% Tris‐glycine gel) using a Novex X‐Cell electrophoresis system (Life Technologies) under constant current (125 mA for 1 hour) and transferred to a methanol activated polyvinylidene difluoride membrane (Immobilon‐P) using the Novex Blot system under constant voltage (100 mA for 90 minutes). Blots were blocked in blocking buffer (PBS, 1% Tween‐20, 5% skimmed milk) for 1 hour at room temperature and then incubated in PBS+1%Tween‐20 + 1% milk with primary antibodies overnight at 4°C. Primary antibodies used were mouse anti‐Bcl‐2 (Dako, Glostrup, Denmark, http://www.dako.com, 1:200), mouse anti‐SQST1/p62 (Abcam, 1:1,000), rabbit anti‐GRP78/BiP (Abcam, 1:1,000), mouse anti‐β‐actin (Sigma, 1:5,000). Horseradish peroxidase (HRP)‐conjugated anti‐mouse IgG or anti‐rabbit IgG (BioRad, Hercules, CA, http://www.bio-rad.com) were used as secondary antibodies, and the signal was visualized using an ECL or ECL Plus detection system (Amersham Pharmacia Biotech, Piscataway, NJ, http://www.amersham.com). The integrated optical density of each band was measured in ImageJ, and expression was normalized to actin levels in the same blot for comparative expression assessment.

For dot blot analysis, a strip of nitrocellulose membrane was used, and 10 µL containing 10 µg of protein were loaded as a dot. The blot was allowed to dry and blocked in 5% skim milk 0.1% Tween/PBS for 1 hour. The blocking buffer was removed and primary antibodies were added to each dot (1:1,000) and incubated for 1 hour at room temperature. The membrane was washed three times for 10 minutes, and HRP‐conjugated anti‐rabbit secondary antibodies were added for 1 hour at room temperature. The membrane was washed three times for 10 minutes in 0.1% Tween/PBS and the signal was detected by ECL (Amersham Pharmacia Biotech).

### Propidium Iodide Staining

Propidium iodide (PI) staining was performed according to the manufacturer's instructions (Sigma). Briefly, the neurons were incubated with 1 µg/mL PI stock solution and RNase A for 10 minutes at 37°C. The solution was then washed with PBS, and cells were fixed in 4% PFA. Immunocytochemistry was then performed as described above.

### Mitochondrial Staining

Cultures of differentiated MNs were incubated with 100 nM of the potentiometric dye MitoTracker Red CMXRos (Thermo Fisher) for 45 minutes at 37°C. The neurons were then washed with PBS, fixed in 4% PFA, and immunostained for Islet1 and SMI‐32 according to the immunocytochemistry protocol above.

### ER Calcium Imaging

Mature MNs were loaded with 10 μM Fluo‐4 AM dissolved in 0.2% dimethyl sulfoxide (Sigma‐Aldrich, St. Louis, http://www.sigmaaldrich.com) with 0.04% Pluronic acid in Hank's Balanced Salts Solution (HBSS) supplemented with CaCl_2_ and MgCl_2_. To image Ca^2+^ signals, we used wide‐field fluorescence microscopy using a Nikon TE 2000 inverted microscope equipped with a ×40 oil‐immersion objective and EMCCD camera (Evolve 128, Photometrics) for imaging fluorescence emission (510–600 nm) at a frame rate of 300 s^−1^. Fluorescence was imaged at 25°C from a 12 × 12 µm (128 × 128 pixel) region within 18 mm coverslips, on which MNs were cultured. ER Ca^2+^ concentration levels were assessed by fluorescence measurements upon application of 1 µM thapsigargin (Life Technologies) in HBSS depleted of CaCl_2_ and MgCl_2_. Fluorescence measurements are expressed as a ratio (Δ*F*/*F*_0_) of the mean change in fluorescence (Δ*F*) at a pixel relative to the resting fluorescence at that pixel before stimulation (*F*_0_). Image analysis was performed using MetaMorph and ImageJ software.

### RNA Fluorescence In Situ Hybridization (FISH)

Cells grown on 12 mm RNase‐free coverslips in 24‐well plates were fixed using 4% PFA for 20 minutes, washed in PBS, and permeabilized using 0.2% Triton X‐100 (Sigma) in PBS for 30 minutes. Cells were then dehydrated in a graded series of alcohols, air dried, and rehydrated in PBS, briefly washed in 2× standard sodium citrate (SSC) and then incubated in prehybridization solution and hybridization solution as previously described using Cy3‐conjugated 2′*O*‐methyl RNA probes (Integrated DNA Technologies) 35. Probe sequences were (CCCCGG)_4_ or (CAGG)_6_. Optional treatments using RNase (0.1 mg/mL; Life Technologies) or DNase (150 U/mL, Qiagen) were performed at 37°C for 1 hour following permeabilization. Following hybridization washes, staining with Tuj1 (1:2000, Covance) and Islet1 (1:200, DSHB) were performed as described above.

### Electrophysiology

A coverslip containing iPSC‐derived MNs was placed in a recording chamber mounted onto the stage of an upright microscope and the somata were targeted for recording using IR‐DIC optics. Cells were continuously superfused with oxygenated artificial cerebrospinal fluid aCSF (95% O_2_/5% CO_2_) containing 130 mM NaCl, 25 mM NaHCO_3_, 2.5 mM KCl, 1.25 mM NaH_2_PO_4_, 2 mM CaCl_2_, 2 mM MgCl_2_, and 10 mM glucose. Patch‐clamp electrodes (4–7 MΩ) were filled with an intracellular solution containing 120 mM K‐gluconate, 10 mM KCl, 10 mM HEPES, 4 mM MgATP, 0.3 mM NaGTP, 10 mM Na‐phosphocreatine. Recordings were obtained using a Multiclamp 700B amplifier and digitized at 10–20 kHz using Digidata 1550 acquisition board.

### Southern Blotting

5 µg of DNA were digested with 2 µL each of FastDigest DdeI and FastDigest AluI (Thermo Scientific) for 2 hours at 37°C. Digested DNA underwent electrophoresis in 0.8% agarose gel in ×1 Tris‐acetate‐EDTA (TAE) and was transferred to a positively charged nylon membrane (Roche, Basel, Switzerland, http://www.roche-applied-science.com) by capillary blotting. Roche digoxigenin (DIG)‐labeled DNA molecular weight marker II was used as a ladder. DNA was crosslinked to the membrane by UV irradiation. Prehybridization was carried out in 20 mL DIG Easy Hyb solution (Roche) at 53°C for 2 hours. 10 ng DIG‐labeled (GGGGCC)_5_ probe (custom synthesized by Integrated DNA Technologies) was incubated at 95°C for 5 minutes, snap cooled on ice for 30 seconds, and immediately added to 7 mL DIG Easy Hyb solution prewarmed to 60°C. Hybridization took place overnight at 53°C. Stringency washes were performed at 64°C as follows: 2× SSC, 0.1% sodium dodecyl sulphate (SDS) for 5 minutes, 2× SSC, 0.1% SDS for 10 minutes, 1× SSC, 0.1% SDS for 15 minutes three times, 0.5× SSC, 0.1% SDS for 15 minutes twice.

The membrane was prepared for detection using the DIG Wash and Block buffer set (Roche). The membrane was washed with wash buffer for 2 minutes then incubated with blocking solution for 1–2 hours. Anti‐DIG‐AP Fab fragments (Roche) were incubated with the membrane at room temperature for 20–25 minutes at 1 in 10,000 dilution in blocking solution. Three washes were carried out for 5, 15, and 15 minutes followed by a 5‐minute equilibration step using the supplied Detection buffer. CDP‐Star (Roche) was added to the membrane for at least 5 minutes, and signal was detected by exposure to x‐ray film. Repeat number was estimated by interpolating a plot of log_10_ of band size in base pairs versus distance travelled on the blot, with subtraction of 199 bp from the estimated base‐pair number to account for the wild‐type allele as performed by Beck et al. 36.

### qRT‐PCR

RNA was isolated from iPSC‐derived MNs using the Qiagen RNeasy kit, according to the manufacturer's instructions. 500 µg of total RNA was used for reverse transcription reaction using SuperScript III reverse transcriptase (Life Technologies). Quantitative real‐time PCR was performed with 100 µg of RNA reverse transcribed to cDNA and SYBR Green qPCR Supermix (Roche). Primers used for qPCR are listed below in Table 1.

**Table 1:** Oligos used for quantitative reverse transcriptase polymerase chain reaction

Oligo	Sequence
BAK_fwd	5′‐GGGTCTATGTTCCCCAGGAT‐3′
BAK_rev	5′‐GCAGGGGTAGAGTTGAGCA‐3′
Bcl‐XL_fwd	5′‐ATGAACTCTTCCGGGATGG‐3′
Bcl‐XL_rev	5′‐TGGACTCAAGGCTCTAGGTG‐3′
BIM_fwd	5′‐GGTCCTCCAGTGGGTATTTCTCTT‐3′
BIM_rev	5′‐ACTGAGATAGTGGTTGAAGGCCTGG‐3′
HPRT_fwd	5′‐GCTATAAATTCTTTGCTGACCTGCTG‐3′
HPRT_rev	5′‐AATTACTTTTATGTCCCCTGTTGACTGG‐3′

### Repeat‐Primed PCR

Repeat‐primed PCR (RP‐PCR) assay was performed as previously described 1, in a reaction volume of 15 μL, containing 100 ng genomic DNA, 8 μL Extensor mastermix (Thermo Scientific), 6 μL of 5 M Betaine, 20 μM of both forward and repeat specific primer and 2 μM of reverse primer. The reactions were subjected to touchdown PCR programme, then 1 μL of each PCR product was mixed with 8 μL of Formamide, and 0.5 μL GeneScan size standard (Applied Biosystem, Foster City, CA, http://www.appliedbiosystems.com). Then samples were analyzed on an ABI3730 DNA Analyzer and visualized using GeneMapper software. Primer sequences used: RP1‐F FAM‐TGTAAAACGACGGCCAGTCAAGGAGGGAAACAACCGCAGCC; RP1‐R CAGGAAACAGCTATGACC; RP1‐Repeat: CAGGAAACAGCTATGACCGGGCCCGCCCCGACCACGCCCCGGCCCCGGCCCCGG.

### Electron Microscopy

Cells grown on polyester filters were immersion fixed with 2.5% glutaraldehyde in 0.1 M phosphate buffer (pH 7.2) for 10 minutes at room temperature and prepared for electron microscopy (EM) by standard methods. Briefly, filters were stained with osmium tetroxide (1% v/v in 0.1 M phosphate buffer) and uranyl acetate (2% w/v in distilled water), dehydrated through increasing concentrations of ethanol (70%–100%) and acetone and embedded in Spurr's resin (Agar scientific, Reading, U.K.). Ultrathin sections (50–80 nm) were prepared by use of a Reichart‐Jung ultracut microtome and mounted on nickel grids (Agar Scientific, Stanstead, Essex, U.K.). Sections were counterstained with lead citrate and uranyl acetate and examined on a JOEL 1010 transmission electron microscope (JOEL USA, Peabody, MA).

### Statistical Analyses

All quantitative data were analyzed using GraphPad Prism. Groups of three or more samples were subject to one‐way ANOVA, with Bonferroni or Dunnett's post hoc comparisons. Alpha was set at 0.05 for all ANOVAs and post hoc tests.

### Dataset Deposit

The Illumina HT12v4 transcriptome array results have been deposited in the GEO under accession number GSE64583. SNP datasets have been deposited in GEO under the accession number GSE64582. These are both contained in the Superseries GSE64584.

## Results

### Reprogramming of Patient Fibroblasts to Generate iPSCs

**Figure 1:**
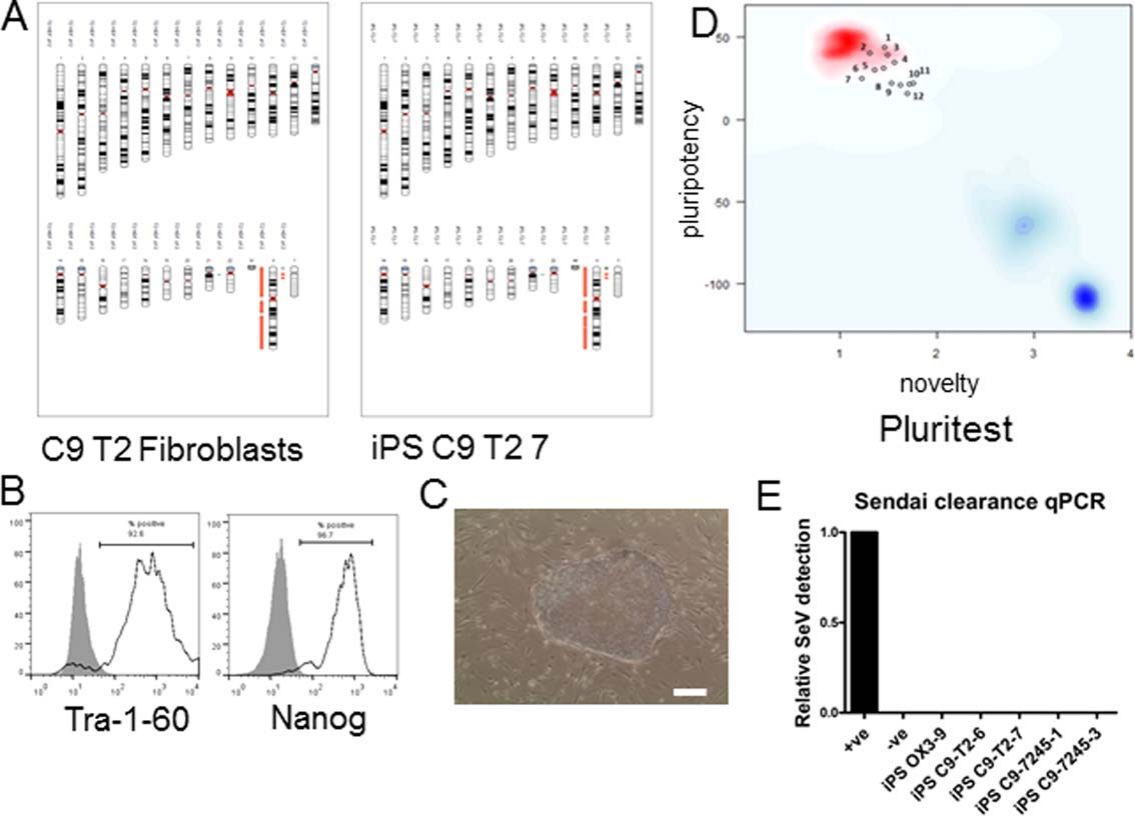
iPSC line characterization. Characterization data for line iPS C9 T2 7. **(A)**: Karyogram showing genome integrity of iPSC (Illumina SNP array data analyzed using Karyostudio). Autosomal detected regions deviating from reference data are annotated with green (amplification) or orange bands (deletion); the left hand panel shows the parental fibroblasts as reference. **(B)**: FACS analysis of iPSC for pluripotency markers Tra‐1‐60 and Nanog (black line); grey filled plot, isotype control. **(C)**: Human iPSC show pluripotent stem cell‐like morphology (cell‐cell‐contact‐dependent clusters, with high nucleus to cytoplasm ratio) by phase microscopy; scale bar = 100 µm. **(D)**: PluriTest analysis of Illumina HT12v4 transcriptome array data shows that all the iPSC lines used cluster with pluripotent stem cells (red cloud) and not with partly‐ or differentiated cells (blue clouds). Each circle represents one iPSC line, with previously published lines OX1‐19 and NHDF‐1 included as reference points [1 = AH017‐13; 2 = OX3‐9; 3 = OX1‐61; 4 = C902‐10; 5 = AH017‐3; 6 = OX1‐19; 7 = NHDF‐1; 8 = 7245‐1; 9 = 7245‐3; 10 = T2‐7; 11 = C902‐02; 12 = T2‐6]; *y* axis pluripotency score, *x* axis novelty score. **(E)**: Assessment of Sendai clearance from iPSC lines reprogrammed with SeVdp(KOSM)302L, by qRT‐PCR, relative to positive control. Abbreviations: iPSC, induced pluripotent stem cell; qPCR, quantitative polymerase chain reaction

Two lines from each of three patients (C9‐T2, C9‐7245, C9‐02) were generated, along with lines from control donors. Figure 1 shows the representative characterization data for one of the patient iPSC lines generated for this study, iPSC C9‐T2‐7 (data for the other previously unpublished iPSC lines, including control lines, is given in Supporting Information Fig. S1). SNP analysis confirmed the identity of the iPSC in relation to their parental fibroblasts and that they had not acquired large‐scale copy number variations, although a small number of small‐scale indels (well below the level that would be detected by G‐banding) were noted, particularly in the C9‐7245 lines (Fig. 1A; Supporting Information Fig. S1A). SNP datasets for these lines have been deposited in GEO under the accession number GSE64582.iPSC lines were positive for pluripotency markers (TRA‐1‐60 and Nanog, Fig. 1B; also Supporting Information Fig. S1B) and showed human pluripotent stem cell‐like morphology (cell‐cell‐contact‐dependent clusters, with high nucleus to cytoplasm ratio, Fig. 1C; Supporting Information Fig. S1C). PluriTest is a quantitative assessment of pluripotency based on transcriptome data from iPSCs in comparison to a large reference set of genome‐wide profiles from multiple cell and tissue types. It measures a “pluripotency score” (expression of pluripotency genes, with high scores correlating with high pluripotency, *y*‐axis) and a “novelty” score (expression of nonpluripotent stem cell genes, therefore this score inversely correlates with pluripotency, *x*‐axis). All the patient iPSC lines were deemed to be fully reprogrammed and pluripotent by this analysis (Fig. 1D). The Illumina HT12v4 transcriptome array results have been deposited in the GEO under accession number GSE64583. Finally, all banked iPSC line stocks had undetectable levels of residual Sendai virus RNA by qRT‐PCR (Fig. 1E; Supporting Information Fig. S1D).

### *C9orf72* iPSCs Differentiate to Functional MNs

In vitro cell differentiation protocols recapitulate signaling events that occur during embryogenesis. The differentiation of iPSCs to spinal MNs is a three‐stage process that involves neuralization, caudalization, and ventralization. The iPSC lines were differentiated to MNs according to three previously published protocols, with some modifications 29, 30, 33 (Supporting Information Fig. S2A–S2C).

The fully differentiated MNs were identified by immunostaining with the MN marker HB9 and neuronal marker β‐III tubulin (Tuj1), which indicated successful differentiation of iPSCs to MNs (Supporting Information Fig S2D). The iPSC‐derived MNs showed expression of synaptophysin, a synaptic vesicle protein essential for the formation of synapses, as well as the expression of choline acetyltransferase, which demonstrates an advanced degree of cholinergic neuron maturation (Supporting Information Fig S2D, S2E).

**Figure 2:**
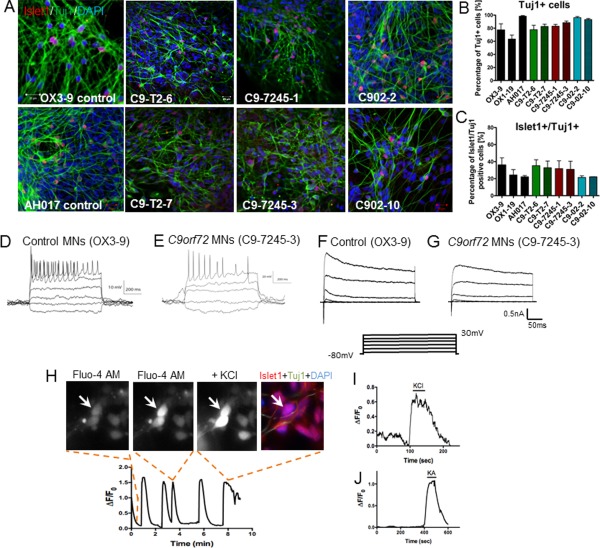
Functional characterization of *C9orf72* induced pluripotent stem cell (iPSC)‐derived MNs. **(A)**: Immunostaining for Islet1 and Tuj1 in control lines (OX1‐19, OX3‐9, AH017) and six patient lines (C9‐T2‐6, C9‐T2‐7, C9‐7245‐1, C9‐7245‐3, C902‐2, C902‐10). **(B)**: Quantification of Tuj1 positive cells shows 80%–95% of cells are neuronal and **(C)** quantification of Islet1 positive cells shows 23%–40% of cells are motor neurons, with nonsignificant differences between the genotypes (*, *p* > .05, one‐way ANOVA). **(D)**: Whole cell recording reveals that trains of action potentials can be evoked reliably in both control MNs and **(E)** *C9orf72* MNs. Representative voltage clamp data showing inward and outward currents recorded during step membrane depolarization of control OX3‐9 **(F)** and C9‐7245‐3 **(G)** neurons. **(H)**: Spontaneous calcium oscillations were recorded from neurons after incubation with Fluo‐4 AM, and typical elevations in intracellular calcium were detected on stimulation with **(I)** KCl and (H) kainate (control neurons are shown in this image). *n* = 3 independent differentiations; *m* =10–15 neurons recorded per line; 5 neurons per line for ionic current measurements. Data are represented as average ± SEM. Scale bar = 20 µm. Abbreviations: DAPI, 4′,6‐Diamidino‐2‐Phenylindole; MNs, motor neurons.

Two iPSC lines were reprogrammed from each patient's fibroblasts and differentiated to MNs for characterization. To assess differentiation efficiency, MNs positive for Islet1/Tuj1 were counted in control lines AH017‐13, OX3‐9, OX1‐19, and in *C9orf72* patient lines C9‐T2‐6, C9‐T2‐7, C9‐7245‐1, C9‐7245‐3, C902‐2, and C902‐10 (Fig. 2A). Differentiation efficiency was assessed after 8 weeks of neural culture, with a highly neuralized population indicated by a mean of 82% of Tuj1‐positive cells across all lines (Fig. 2B). Approximately half of the Tuj1‐positive neural cells in these cultures were also positive for the postmitotic MN marker Islet1 (42.7%–46.4% of total cells) (Fig. 2C). CNs were differentiated in parallel, and functional experiments were carried out after 70–100 days in culture 34 (Supporting Information Fig. S2E). The sizes of the expansions were determined by Southern blotting in the mature MNs and estimated at 510–690 repeats for C9‐T2‐6, 420–640 repeats for C9‐T2‐7, 1,210 for C9‐7245‐1, 1,380 for C9‐7245‐3, 1,000 for C902 (Supporting Information Fig. S3A). The presence of the repeats was also confirmed by RP‐PCR in C9‐02‐2 and C9‐7245‐3 MNs and the absence of repeats was confirmed in the control lines OX1‐19 and AH017‐13 (Supporting Information Fig. S3B).

To assess the functional maturation of the iPSC‐derived MNs, we performed whole‐cell patch clamp recordings to determine their electrophysiological properties. Trains of action potentials were elicited in both control and patient MNs in response to depolarizing current injection in current‐clamp recordings (Fig. 2D, 2E). Inward and outward ionic currents were recorded by voltage clamp in both controls and patients (Fig. 2F, 2G). Ca^2+^ dynamics were investigated using the calcium‐sensitive dye Fluo‐4 AM. In the absence of external stimulation, spontaneous calcium transients were detectable in the cell bodies and processes of the neurons (Fig. 2H). The neurons were then stimulated with kainate (KA), to activate ionotropic receptors, and with KCl to depolarize the membrane and activate voltage‐gated Ca^2+^ channels. Exposure to KCl induced Ca^2+^ transients in Islet1+ MNs (Fig. 2I). Similarly, exposure to KA induced characteristic increases in intracellular Ca^2+^ levels in 80% of the cells (Fig. 2J).

### RNA Foci and RAN Dipeptides are Present in *C9orf72* MNs

**Figure 3:**
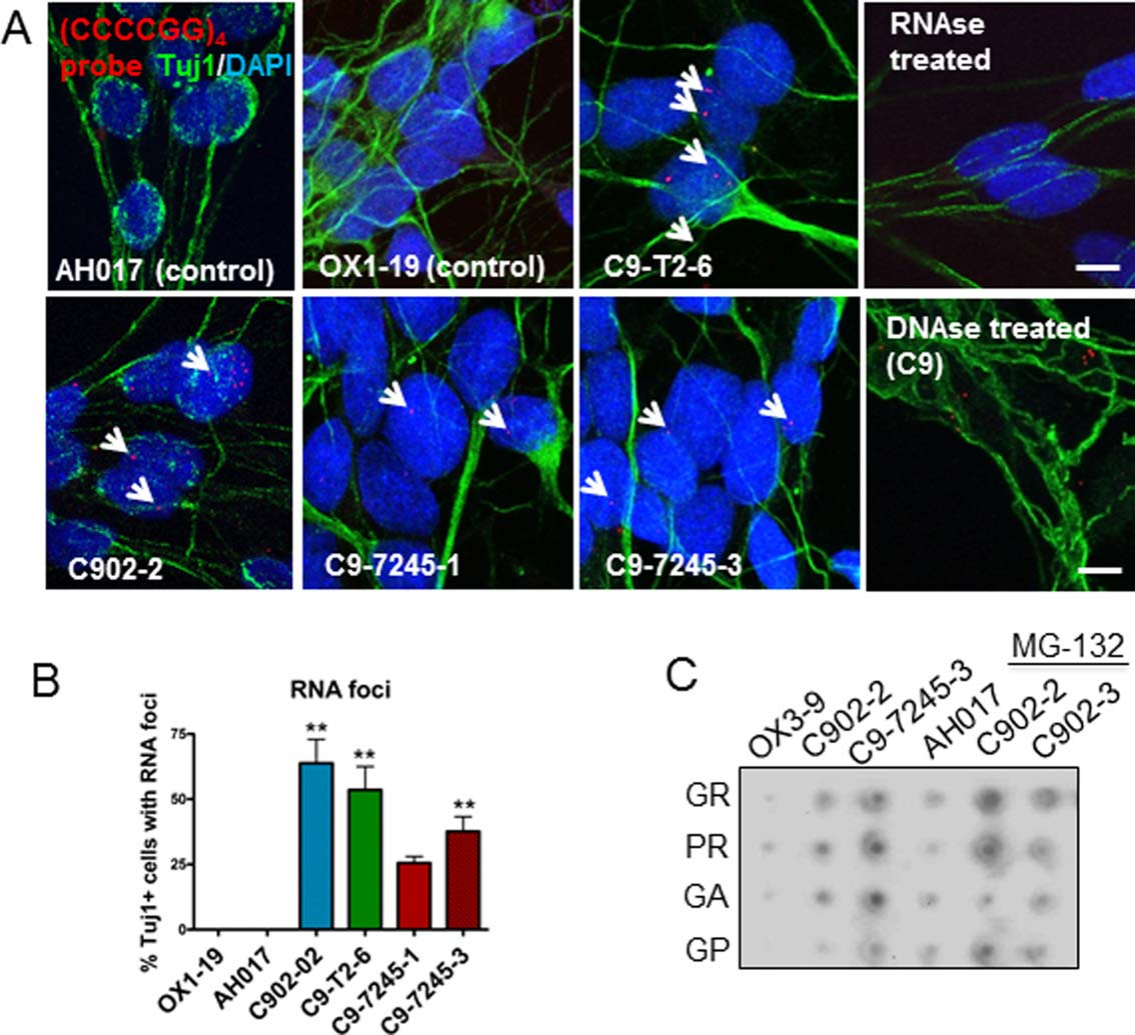
RNA foci and repeat‐associated non‐ATG dipeptides are detected in *C9orf72* patient‐derived motor neurons (MNs). **(A)**: [CCCCGG]_4_ probe was used to detect sense RNA foci from the *C9orf72* hexanucleotide expansions. **(B)**: RNA foci were detected in up to 65% of neurons from *C9orf72* induced pluripotent stem cell (iPSC)‐derived MNs (121–157 neurons counted), while no foci were detected in the controls (**, *p* < .01, one‐way ANOVA). **(C)**: Dot blot analysis of GR, PR, GA, and GP dipeptides in controls (OX3‐9 and AH017) and patient lines (C902‐2, C9‐7245‐3 and C902‐3) at baseline and after treatment with MG‐132 for 24 hours. Scale bar = 5 µm. Data are represented as average ± SEM. Abbreviation: DAPI, 4′,6‐Diamidino‐2‐Phenylindole.

In the MNs derived from *C9orf72* patients, we identified a significant increase in intranuclear GGGGCC‐repeat containing RNA foci in all lines (Fig. 3A, 3B). The specificity of the probe was confirmed by treatment with RNase, which reduced the number of foci substantially (Supporting Information Fig. S4A), and with DNase I, after which the number of foci was maintained. To investigate evidence for the RAN translation hypothesis, we performed a dot blot analysis using anti‐GA, GP, GR, and PR antibodies, and we could detect traces of all the dipeptides in the MNs from *C9orf72* patients (Fig. 3C).

### Elevated ER Ca^2+^ Levels, Altered Mitochondrial Morphology, and Reduced Bcl‐2 Levels in *C9orf72* MNs

**Figure 4:**
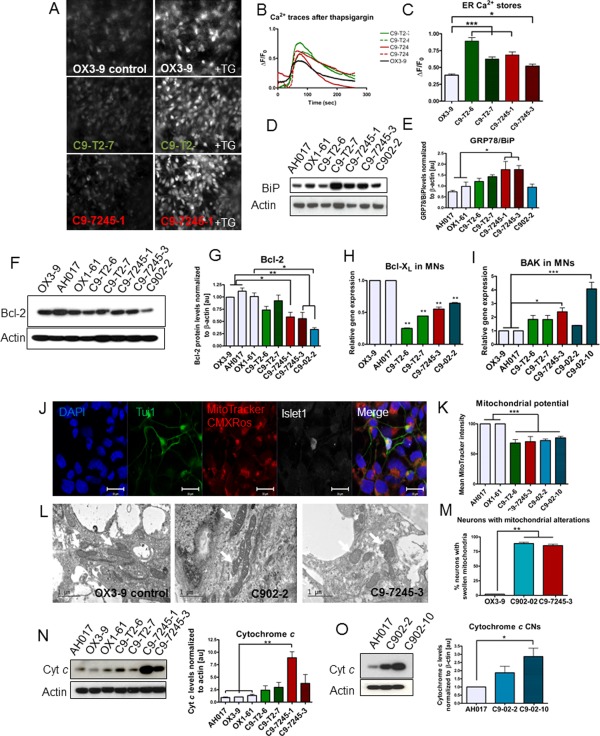
Increased ER Ca^2+^ content is detected in *C9orf72* induced pluripotent stem cell (iPSC)‐derived MNs along with mitochondrial alterations and Bcl‐2 protein imbalance. **(A)**: Images representing the iPSC‐derived neurons before and after TG stimulation and **(B)** their representative fluorescent traces. **(C)**: Measurement of the TG‐evoked ER Ca^2+^ release shows significantly higher ER Ca^2+^ levels in C9‐T2 and C9‐7245 lines compared to OX3‐9 (***, *p* < .001; *, *p* < .05; one‐way ANOVA with Dunnet's post hoc test); *n* = 3 independent differentiations, *m* = 150–200 neurons recorded per genotype. **(D)**: Immunoblotting shows elevated levels of GRP78/BiP in all patient lines and significantly upregulated in C9‐7245‐1 and C9‐7245‐3 (*, *p* < .05, one‐way ANOVA with Dunnet's post hoc test) **(E)**. *n* = 3 independent differentiations, *m* = 142–200 neurons recorded per genotype per differentiation. **(F)**: Immunoblotting for the antiapoptotic Bcl‐2 protein shows downregulation of levels in patient lines and **(G)** significant reduction in C9‐7245‐1 and C9‐7245‐3 and C902‐2 compared to control OX3‐9, AH017‐13, and OX1‐61 (*, *p* < .05; **, *p* < .01, one‐way ANOVA with Dunnett's post hoc test); *n* = 3 independent differentiations (*n* = 2 independent differentiations for AH017‐13 and OX1‐61), *m* = 3–4 technical replicates. **(H)**: Quantitative polymerase chain reaction confirms downregulation of Bcl‐X_L_ and upregulation of **(I)** BAK, specifically in the C9‐7245 and C902‐2 MN lines (*, *p* < .05; **, *p* < .01; ***, *p* < .001, one‐way ANOVA with Dunnett's post hoc test); *n* = 2 independent differentiations; *m* = 3 technical replicates. **(J)**: Representative image of OX3‐9 loaded with MitoTracker Red CMXRos and immunostained with Islet1 and Tuj1. **(K)**: Quantification of the mean fluorescence intensity of MitoTracker in Islet1^+^/Tuj1^+^ neurons shows reduced mitochondrial membrane potential in all patient lines (***, *p* < .001, one‐way ANOVA with Dunnett's post hoc test); *n* = 2 independent differentiations; *m* = 17–31 neurons analyzed per genotype. **(L)**: Electron micrographs (EM) show enlarged mitochondria and christae in the patient lines C902‐2 and C9‐7245‐3 compared to a healthy control (OX3‐9). Arrows point to mitochondria. **(M)**: Quantification of EM images for mitochondrial alterations show 88% of *C9orf72* neurons with swollen mitochondria (**, *p* < .01, ANOVA with Dunnet's post hoc test). **(N)**: Cytochrome *c* levels are elevated in all patient lines and significantly higher than healthy controls in C9‐7245‐1 (*, *p* < .05, one‐way ANOVA with Dunnett's post hoc test). *n* = 2 independent differentiations, *m* = 2 technical replicates each. **(O)**: CNs show elevated levels of the apoptotic marker cytochrome *c* in C902‐10 (*, *p* < .05; **, *p* < .01, one‐way ANOVA with Dunnett's post hoc test). *n* = 2 independent differentiations. Scale bars = 20 µm (J), 1 µm (L). Data are represented as average ± SEM. Abbreviations: CNs, cortical neurons; ER, endoplasmic reticulum; MNs, motor neurons; TG, thapsigargin.

Although a direct link between calcium signaling and *C9orf72* expansions has not yet been reported, calcium dysregulation is an established feature of the pathogenesis of ALS. The levels of Ca^2+^ stores in the ER were analyzed in fully differentiated MNs from eight separate cultures of three independent differentiations for each cell line (OX3‐9, C9‐T2‐6, C9‐T2‐7, C9‐7245‐1, and C9‐7245‐3). To assess the total content of ER Ca^2+^ stores, 10 μM thapsigargin (TG) was applied to the MNs, and the peak increase in fluorescence intensity resulting from ER Ca^2+^ depletion into the cytoplasm was measured (Fig. 4A, 4B). TG acts as a potent inhibitor of the SERCA pump on the ER membrane, blocking the reuptake of Ca^2+^ ions from the cytoplasm. In our cultures, we detected significantly higher levels of ER Ca^2+^ content in the *C9orf72* iPSC‐derived MNs, which showed up to twofold increase in cytoplasmic fluorescence intensity compared to the control MNs (*p* < .05, one‐way ANOVA) (Fig. 4C).

Abnormal elevations in ER Ca^2+^ levels can cause ER stress and can activate the UPR. We investigated levels of proteins associated with the UPR and ER stress responses, and we found significantly elevated levels of GRP78/BiP in C9‐7245‐1 and C9‐7245‐3 iPSC‐derived MNs compared to AH017‐13 and OX1‐61 MNs (*p* < .05, one‐way ANOVA) (Fig. 4D, 4E). We did not detect differences in the splicing of XBP‐1 between patients and controls (Supporting Information Fig. S5A).

The Bcl‐2 family of proteins are key components involved in the modulation of calcium homeostasis in the ER, controlling the release of Ca^2+^ ions through the IP_3_R and through channels formed by its own dimerization 19. The balance between proapoptotic and antiapoptotic members of the Bcl‐2 family is essential for controlling ER stress‐induced apoptosis and mitochondrial membrane permeability 17. In *C9orf72* MNs, we detected an imbalance between proapoptotic and antiapoptotic members of the Bcl‐2 family, which showed up to 50% downregulation of antiapoptotic Bcl‐2 in C9‐7245‐1, C9‐7245‐3, and C902‐2 patient lines (Fig. 4F, 4G) and 75% reduction of Bcl‐X_L_ (*p* < .05, one‐way ANOVA) (Fig. 4H). Significant upregulation of proapoptotic BAK was detected in the MNs with large expansions, which showed up to 2.5‐fold increase in expression (*p* < .05, one‐way ANOVA) (Fig. 4I). A significant upregulation of the proapoptotic BIM was also detected in some of the MN lines (Supporting Information Fig. S5B).

The Bcl‐2 family of proteins is distributed throughout the ER and mitochondrial membranes, where they play critical roles in regulating intracellular Ca^2+^ homeostasis and exchange of Ca^2+^ between the two organelles. Following dysfunction of these proteins and Ca^2+^ overload, the mitochondria undergo typical morphological changes, such as swelling and perturbation of the outer membrane, accompanied by the release of apoptotic factors 37. To assess these alterations, we used the potentiometric dye MitoTracker Red CMXRos, accumulation of which is dependent on mitochondrial potential (Fig. 4J). We found up to 30% reduction in mitochondrial membrane potential in C9 MNs compared to controls (*p* < .01, one‐way ANOVA) (Fig. 4K).

In line with these findings, analysis of morphological alterations of the mitochondria using EM also revealed swollen mitochondria in the neurons from *C9orf72* patients compared to the healthy control (Fig. 4L). Alterations of the mitochondrial morphology and abnormalities in the cristae structures were observed in up to 88% of *C9orf72* neurons compared to only 2% normal healthy neurons (Fig. 4M).

These mitochondrial alterations are consistent with the release of apoptotic factors, such as cytochrome *c*, which was found to be elevated in all patient lines, specifically in the C9‐7245‐1 compared to healthy controls (*p* < .05, one‐way ANOVA) (Fig. 4N, 4O). These results indicate an association between *C9orf72* expansions and elevated ER Ca^2+^ concentrations and stress, as well as mitochondrial alterations which may be the drivers for downstream apoptosis.

### *C9orf72* iPSC‐Derived MNs and CNs have Increased Susceptibility to Apoptosis

**Figure 5:**
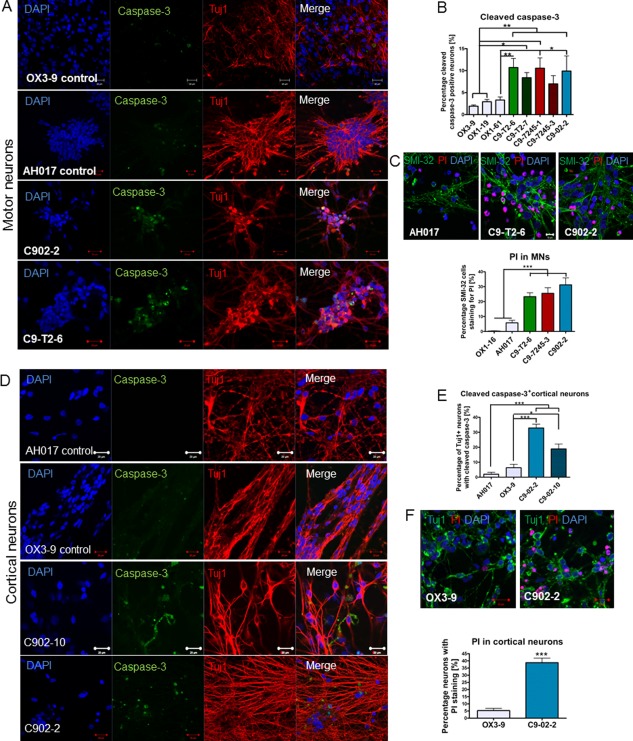
Cleaved caspase‐3 is frequently detected in *C9orf72* MNs and cortical neurons (CNs) and reduced cell viability is found in *C9orf72* MNs and CNs. **(A, B)**: Immunostaining for cleaved caspase‐3 shows increased frequency of apoptotic neurons in *C9orf72* MNs compared to control MNs (*, *p* < .05; **, *p <* .01, one‐way ANOVA with Dunnett's post hoc test); *n* = 3 independent differentiations; *m* = 400 cells per genotype analyzed for each individual experiment. **(C)**: Reduced cell viability was detected in *C9orf72* MNs by PI staining (***, *p* < .001, one‐way ANOVA with Dunnett's *post hoc* test) *n* = 2 independent differentiations. **(D, E)**: Immunostaining with cleaved caspase‐3 shows significantly increased frequency of positive cortical neurons in C902‐2 and C902‐10 lines compared to two healthy control lines (OX3‐9 and AH017‐13) (*, *p* < .05; ***, *p* < .001, one‐way ANOVA with Dunnett's post hoc test). **(F)**: Reduced viability was detected by PI staining in the *C9orf72* patient (***p < .001, one‐way ANOVA with Dunnett's post hoc test). *n* = 3 independent differentiations. Data are represented as average ± SEM. Scale bar = 20 µm. Abbreviations: MNs, motor neurons; PI, propidium iodide.

To investigate whether MNs and CNs carrying pathogenic hexanucleotide expansions in *C9orf72* develop an apoptotic phenotype under normal culture conditions, we assessed markers of cellular stress and cell death. Caspases are present in the cell as inactive proenzymes which can be activated by proteolytic cleavage, the cleavage of caspase‐3 being an essential process that initiates the apoptotic disassembly of the cell. In neurons from *C9orf72* patients we found significantly increased immunostaining for cleaved caspase‐3 compared to the CNs from OX1‐19, OX3‐9, and AH017‐13 (Fig. 5A). We detected between 8.1% and 10.5% of neurons positive for cleaved caspase‐3 in the *C9orf72* patient lines, compared to a maximum of 2.9% in OX1‐19 and 1.9% in OX3‐9 (*p* < .05, one‐way ANOVA) (Fig. 5B). In line with this finding, the ALS/FTD‐derived MNs also showed significantly increased staining with PI in up to 32% of *C9orf72* MNs compared to healthy MNs, indicating reduced survival compared to control MNs (*p* < .01, one‐way ANOVA) (Fig. 5C).

In CN cultures, we found a significantly high frequency of cleaved caspase‐3 in the C902‐2 and C902‐10 lines, with up to 35% of neurons positive for apoptotic markers compared to controls (*p* < .05, one‐way ANOVA) (Fig. 5D, 5E). These results also correlated with high PI staining, which was found in up to 40% of the neurons analyzed in the C902 patient lines (*p* < .001, Student's *t* test) (Fig. 5F). Taken together, these results demonstrate that the presence of the *C9orf72* hexanucleotide expansions activates caspase‐3 dependent apoptosis in both MNs and CNs of patients.

### Stress Granules and Protein Aggregates are Present in *C9orf72* iPSC‐Derived MNs and CNs

**Figure 6:**
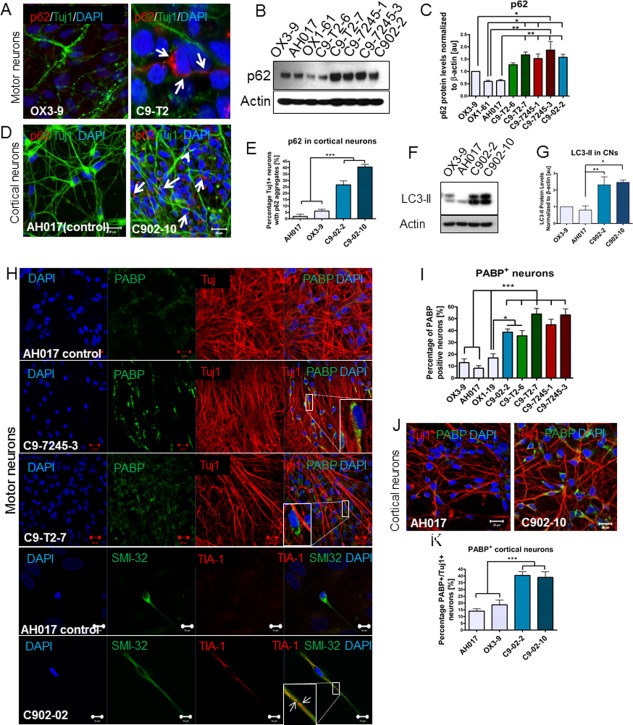
p62 is elevated in *C9orf72* neurons and PABP immunopositive stress granules form spontaneously in *C9orf72* neurons. **(A)**: Immunostaining for p62 in C9 motor neurons (MNs) shows aggregates are formed under baseline conditions. **(B, C)**: Immunoblotting demonstrates high levels of p62 in C9‐T2‐6, C9‐7245‐3, and C902‐2 MNs compared to controls under baseline conditions (*, *p* < .05; **, *p* < .01, one‐way ANOVA with Dunnet's post hoc test); *n* = 2 independent differentiations, *m* = 3 technical replicates. **(D, E)**: p62 aggregates are detected more frequently in C9 CNs compared to controls (***, *p* < .001, one‐way ANOVA with Dunnett's post hoc test). **(F, G)**: Immunoblotting shows elevated levels of LC3‐II in both lines from the *C9orf72* patient C902 compared to controls (***, *p* < .001, one‐way ANOVA with Dunnett's post hoc test); **(H)** PABP^+^ and TIA‐1^+^ stress granules are detected in all five C9 patient lines under baseline conditions and **(I)** PABP^+^ MNs are significantly more frequent than in controls (*, *p* < .05; ***, *p* < .01, one‐way ANOVA with Dunnet's post hoc test); *n* = 3 independent differentiations; *m* = 387–918 neurons analyzed per genotype. **(J, K)**: PABP^+^ stress granules form in C9 CNs (***, *p* < .001, one‐way ANOVA with Dunnett's post hoc test). Scale bars = 20 µm; scale bars in the bottom panels of (H) = 10 µm. Data are represented as average ± SEM. Abbreviations: CNs, cortical neurons; DAPI, 4′,6‐Diamidino‐2‐Phenylindole; PABP, poly‐A‐binding protein.

SQST1/p62, a marker of the UPR and autophagy flux is found in neuronal aggregates in autopsy samples from *C9orf72* ALS/FTD cases. We found significantly elevated levels of SQST1/p62 in MNs derived from each *C9orf72* patient, and the patient with the highest number of repeats showed up to twofold higher expression of SQST1/p62 compared to control MNs at baseline (*p* < .05, one‐way ANOVA) (Fig. 6A–6C). No differences were detected in the autophagic marker LC3‐II/LC3‐I in MNs (Supporting Information Fig. S5C, S5D). In CNs carrying the *C9orf72* expansions we also found approximately 40% neurons positive for p62 aggregates (Fig. 6D–6F). In contrast to MNs, these results correlated with an increase in levels of LC3‐II in C902‐2 and C902‐10 compared to controls OX3‐9 and AH017‐13 (*p* < .05, one‐way ANOVA) (Fig. 6G).

Stress granules are formed when RNA binding proteins aggregate through their glycine rich domains. These granules function to sequester, silence, or degrade specific RNA transcripts as part of a mechanism that adapts patterns of local RNA translation to facilitate the response to cellular stress 38. PABP is a major component of stress granules and serves as a robust marker of the activation of the stress response. In our neuronal culture, we found up to fivefold increase in the frequency of neurons with PABP‐positive granules under resting conditions in all five *C9orf72* patient MN lines compared to controls (*p* < .05, one‐way ANOVA) (Fig. 6H, 6I). The *C9orf72* MNs were also immunopositive for the early stress granule marker, TIA‐1 (Fig. 6H). PABP positive stress granules were detected in up to 40% of *C9orf72* CNs compared to a maximum of 20% control CNs (Fig. 6J, 6K).

## Discussion

The inability to directly study neurons in patients suffering from neurodegenerative disorders has been a significant impediment to the understanding of basic mechanisms, particularly those occurring early in the disease process. Recent developments in stem cell technology have substantially expanded the range of cellular models available in ALS by allowing the direct observation of pathomechanisms in MNs derived from iPSC obtained from fibroblasts harboring the genetic background conferring disease susceptibility.

The mechanisms underlying *C9orf72* toxicity in MNs remain to be elucidated, but possibilities include haploinsufficiency and toxic gain‐of‐function, mediated either by RNA foci or toxic aggregation‐prone polypeptides resulting from RAN translation 1, 10, 39, 40. These potential mechanisms are not mutually exclusive and may each play a role. In this study, we identified several converging pathways that are altered in MNs and CNs from *C9orf72* patients.

RNA foci have been consistently detected in the CNs of patients with hexanucleotide repeats 1, 14, and we detected discrete RNA foci in our MN cultures from all *C9orf72* patient lines. Since the presence and number of RNA foci did not correlate with other phenotypes, the relationship of RNA foci to the pathology we observe in this cellular model requires further investigation. A dot blot analysis using high levels of proteins revealed the presence of the GA, GP, GR, and PR dipeptides in the patient lines, but given their low abundance and difficulty of detection, it is challenging to analyze their contribution to neuronal degeneration in our cellular model. A recent study has shown the formation of stress granules in neuronal cells transfected with poly‐GA, poly‐GP, and poly‐PA, suggesting that RAN translation may be associated with stress granule formation and neurodegeneration 41. In our cultures of *C9orf72* MNs and CNs, we detected the frequent presence of stress granules under baseline conditions. Stress granules are conserved cytoplasmic aggregates of nontranslating messenger ribonucleoprotein complexes, and they are dynamic structures which dissociate when conditions return to normal or they are cleared by autophagy 42. The accumulation of PABP^+^ stress granules suggests impairments in autophagy and proteasomal degradation, and the convergence of these two dysfunctional pathways may partially explain the vulnerability of *C9orf72* MNs and CNs to degeneration.

Calcium is known to exert a complex regulatory role in the ER‐mitochondrial crosstalk, playing an essential part in cell death signaling 43. Bcl‐2 together with Bcl‐X_L_ are located on the ER membrane where they lower the [Ca^2+^]_ER_ by forming Ca^2+^ permeable leakage pores 44. Reduced levels of Bcl‐2 and Bcl‐X_L_ contribute to nonphysiological accumulation of Ca^2+^ ions into the ER due to reduced leakage, and we confirmed elevated levels of ER Ca^2+^ stores in the *C9orf72* MNs. We have recently shown that TDP‐43 pathogenic mutations in mouse MNs and in a human cellular model also interfere with ER Ca^2+^, but with the opposite effect, showing reduced ER Ca^2+^ levels and decreased ER Ca^2+^ release 45.

The tight connection between the ER Ca^2+^ stores and mitochondria allows cells to respond promptly and efficiently to various Ca^2+^ stimuli. However, under pathological conditions, the high levels of Ca^2+^ from the ER will overload the mitochondria and consequently induce mitochondrial swelling, rupture of the outer membrane, and release of apoptotic factors from the mitochondria 37. The antiapoptotic protein Bcl‐2 is located on the mitochondrial membrane where it prevents proapoptotic BAK/BAX to oligomerize and to form channels that allow release of the apoptotic factor cytochrome *c* into the cytosol. Low levels of Bcl‐2 and increased levels of BAK promote the release of cytochrome *c* from the mitochondria triggering the activation of caspases that regulate the induction and execution of apoptosis 46. Consistent with this, we detected high levels of cleaved caspase‐3 and cytochrome *c* in the patient‐derived MNs and CNs, indicating the C9 neurons are undergoing caspase‐dependent apoptosis.

Disruption of ER Ca^2+^ homeostasis also plays a direct role in the induction of ER stress by interfering with the function of Ca^2+^‐sensitive ER chaperones 47. Constitutive activation of ER stress in *C9orf72* MNs, which interestingly appears to be greater in cells with a higher number of hexanucleotide repeats, may contribute to the induction of ER‐stress associated apoptosis. This is consistent with a previous study of *C9orf72*‐patient iPSC‐derived MNs which found upregulation of transcripts involved in UPR activation and ER stress 48. While all MNs carrying repeats showed a survival deficit and an active apoptotic pathway, only the patient‐derived MNs with large hexanucleotide expansions showed ER stress under baseline conditions. These results suggest that ER stress is activated by high numbers of pathogenic repeats but is not the exclusive pathway in the induction of neuronal death. In accordance with our observations, a similar study using SOD1^+/A4V^ iPSC‐derived MNs found elevated ER stress in patient MNs, but treatment with salubrinal or knockdown of XBP‐1 did not rescue the survival deficit, suggesting that ER stress is only one of many components that may be contributing to MN death 48. Selective neuronal vulnerability may involve specific combinations of mutually reinforcing stressors and altered pathways that ultimately converge on neuronal death 49. Consequently, the gradual increase of stress in an age‐dependent fashion in vulnerable MNs may underlie the progression of neurodegeneration in ALS. Taken together, our results highlight the importance of ER Ca^2+^ regulation in different in vivo and in vitro models and support the hypothesis that MN degeneration in ALS is mediated by cell‐type specific impairments in intracellular Ca^2+^ handling 50, 51, 52.

MNs and CNs from *C9orf72* patients also showed high levels of SQST1/p62, which is an adaptor molecule for selective autophagosomal degradation of ubiquitinated targets and its accumulation reflects the inefficiency of protein aggregate removal by the autophagic machinery 53. SQST1/p62 is a common component of protein aggregates found in neurodegenerative diseases, such as Lewy bodies in Parkinson's disease, neurofibrillary tangles in Alzheimer's disease, and in huntingtin aggregates 54, 55, 56. Elevated p62 levels have previously been reported in CNs derived from iPSCs of FTD patients carrying hexanucleotide expansions in *C9orf72*, and it is a pathological marker in post‐mortem analysis from *C9orf72* carriers 5, 7. We detected high SQST1/p62 levels in MNs under basal conditions without changes in LC3‐II/LC3‐I turnover, indicating a reduced autophagic flux in *C9orf72* MNs compared to healthy controls. Interestingly, elevated SQST1/p62 levels were correlated with increased LC3‐II in CNs from an ALS/FTD patient, suggesting a potentially distinct accumulation of autophagosomes in these cells and impairment in the autophagic process, which is specific to the CNs and is not recapitulated in MNs.

## Conclusion

Our study identified several cellular phenotypes that associate with the repeat expansions in *C9orf72* in MNs and CNs and molecular changes that may account for their vulnerability in pathological conditions. We have identified an apoptotic pathway that is activated in the *C9orf72* neurons, which involves ER calcium dysregulation and stress, followed by mitochondrial alterations, the release of apoptotic factors from the mitochondria, and the formation of stress granules. It is unclear where this chain of phenotypes is initiated, whether the presence of toxic RAN dipeptides induces ER stress and dysregulation of calcium, or whether the presence of the RNA foci leads to the recruitment of essential Ca^2+^ regulators transcripts. Further work is currently underway to analyze the molecular mechanism linking the hexanucleotide repeats in *C9orf72* and the neuronal phenotypes. In this study, a high number of repeat expansions (>1,000) correlated with a more severe phenotype in our patient MN cultures, but a more extensive study is required to establish whether there is a correlation between the number of repeats and severity of the cellular phenotypes. Although our observations suggest that the cellular phenotypes observed are robust enough to be used to as a tool to screen potential therapies, further work is required to establish the exact mechanistic relationship between the pathological pathways activated in *C9orf72* MNs, CNs, and neurodegeneration.

## Author Contributions

R.D.: conception and design, collection and assembly of data, data analysis and interpretation, manuscript writing, and final approval of manuscript; J.S. and M.N.: differentiation, RNA fluorescence in situ hybridization; N.A.: stem cell reprogramming and iPS expansion and characterization; T.L. and G.W.: electrophysiology experiments; H.C.: electron microscopy data collection and interpretation; J.V.: stem cell reprogramming and iPS expansion; A.G.L.D.: Southern blotting; A.F.‐J.: partial collection of survival data; C.B.: stem cell reprogramming; M.N.: provision of reprogramming factors; M.R.T.: provision of patients; R.W‐M.: supervision of ER Ca^2+^ study; S.A.C.: collection and assembly of data and final approval of manuscript; K.T.: conception and design, generation of funding, provision of study patients, manuscript writing, editing, and final approval of manuscript.

## Disclosure of Potential Conflicts of Interest

The authors indicate no potential conflicts of interest.

## Supporting information

Supplementary Information Figures.
