# Supplementary material for: C9orf72 Hexanucleotide Expansions Are Associated with Altered Endoplasmic Reticulum Calcium Homeostasis and Stress Granule Formation in Induced Pluripotent Stem Cell‐Derived Neurons from Patients with Amyotrophic Lateral Sclerosis and Frontotemporal Dementia
Source: Stem Cells. 2016 May 4;34(8):2063–78. doi: 10.1002/stem.2388 (PMC4979662; doi:10.1002/stem.2388)
Supplement: Supplementary file 1 — Supplementary Information Figures. [file STEM-34-2063-s001.doc]

**Supplementary figures**

Dafinca et al, C9orf72 hexanucleotide expansions are associated with altered ER calcium homeostasis and stress granule formation in iPSC-derived neurons from patients with amyotrophic lateral sclerosis and frontotemporal dementia

**
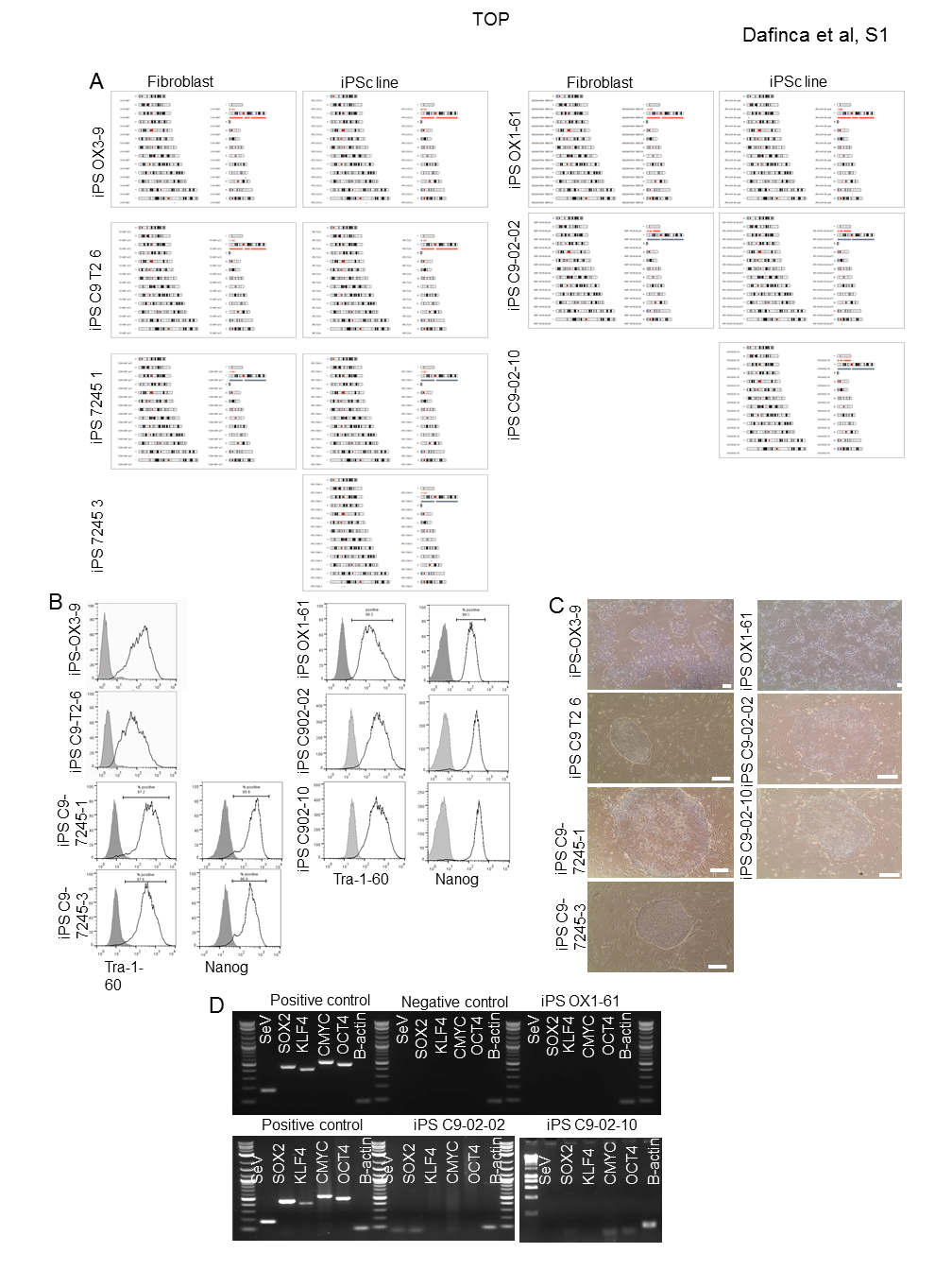
**

**Figure S1. Characterisation of additional iPSC lines**

Characterisation of the remaining iPSc lines used in this study that have not been previously published (see Figure 1 for characterisation of iPS C9 T2 7). A) Karyograms showing genome integrity of iPSc (Illumina SNP array data analysed using Karyostudio). Autosomal detected regions deviating from reference data are annotated with green (amplification) or orange bands (deletion); parental fibroblasts also shown as reference. B) FACs analysis of iPSc for pluripotency markers Tra-1-60 and Nanog (black line); grey filled plot, isotype control. C) Pluripotent stem cell-like morphology (cell-cell-contact-dependent clusters, with high nucleus to cytoplasm ratio) by phase microscopy (iPS-OX3-9 and OX1-61 photographed growing feeder-free; other lines photographed as established colonies in the presence of feeders); scale bar: 100 µm. D) Clearance of Sendai viruses from iPSc lines reprogrammed with Cytotune, as assessed by PCR. Product sizes: SeV backbone 181bp; SeV-Sox 451bp; SeV-Klf 410bp; SeV-Myc 532bp; SeV-Oct 483bp; Actin 92bp.


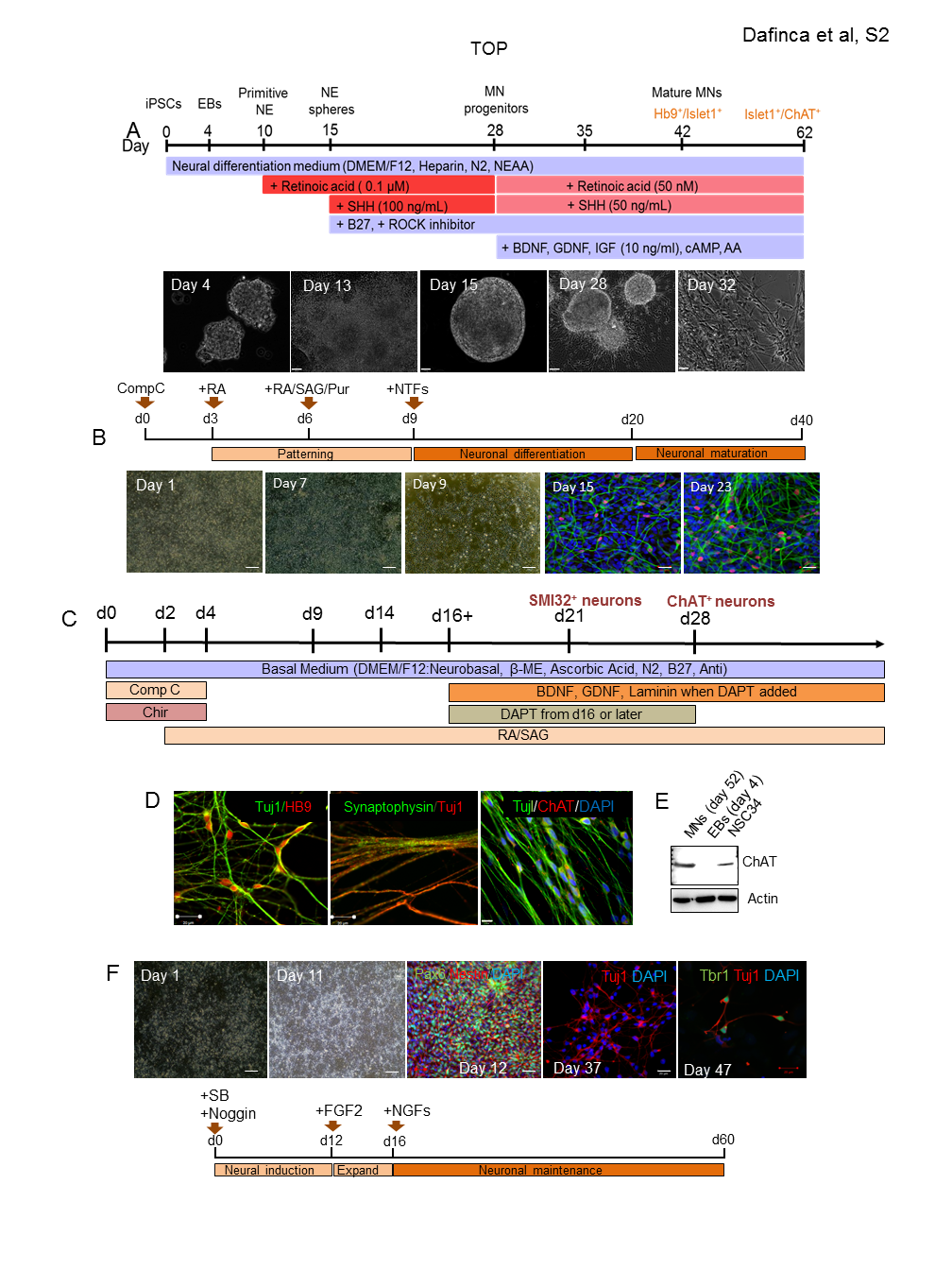


**Figure S2: Differentiation of healthy iPSCs to motor neurons.** A) Schematic representation of the motor neuron differentiation protocol used and representative images of the typical morphology throughout the differentiation (NHDF-1 line is shown). B) Schematic representation of the second motor neuron differentiation protocol used and images of the typical morphology throughout the protocol (AH017-13 line is shown). C) Schematic representation of the third differentiation protocol used in the study. D) Immunostaining for Hb9/Tuj1, Synaptophysin and ChAT confirmed the motor neuron identity of the population after 62 days in culture. E) Immunoblotting for ChAT. F) Schematic representation of the cortical neuron differentiation protocol. Scale bar = 10 µm (for the first 3 images of panels B and F); 20 µm (for the final 2 images of panels B and F).

**
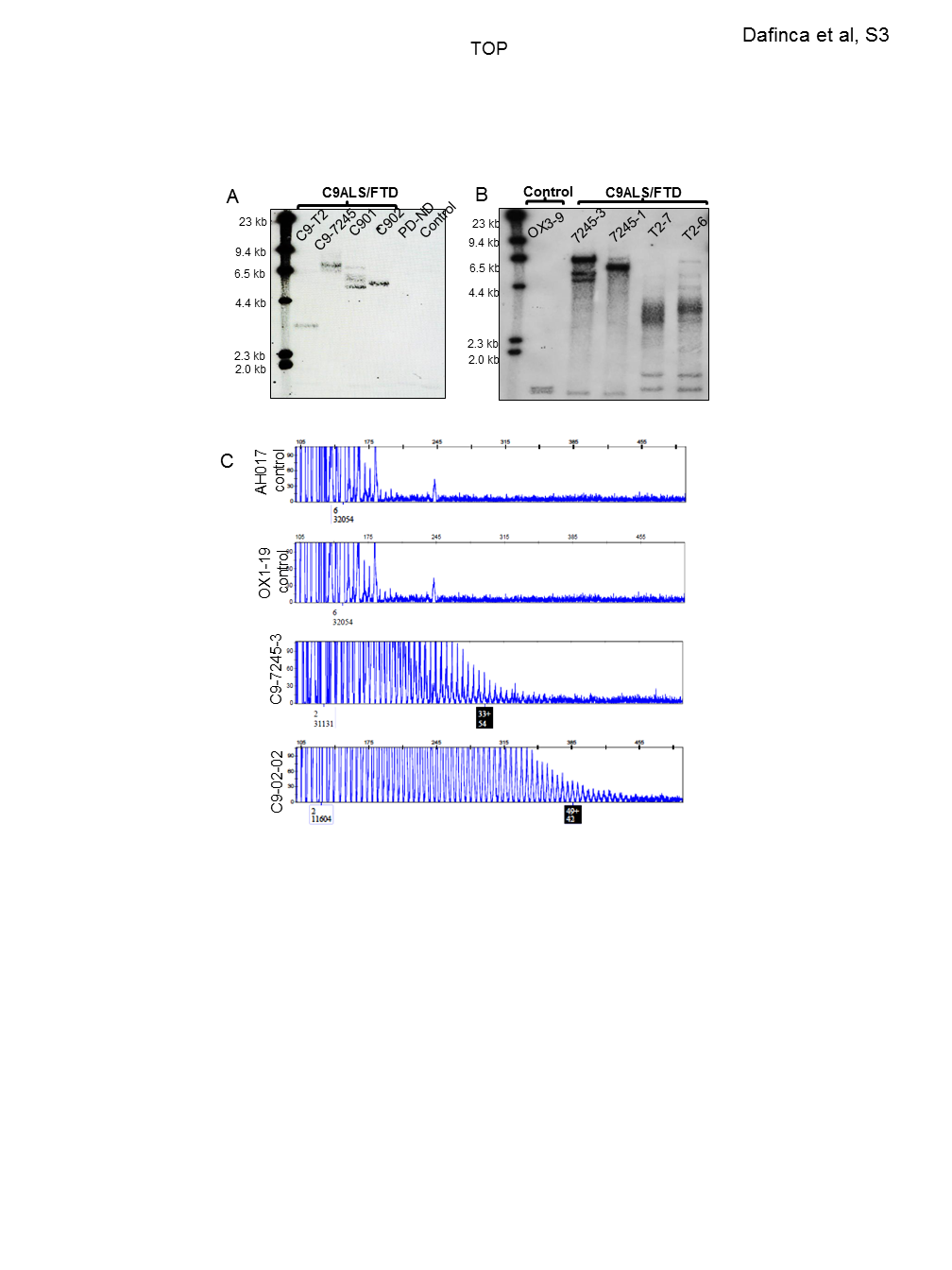
**

**Figure S3. Repeat expansions in *C9orf72* iPSC-derived MNs.** A) Southern blotting shows the presence of the expansions in the C9 fibroblasts used in the study (T2, 7245, C902) compared to a Parkinson’s Disease patient (PD-ND) and healthy control (Control). B) Southern blotting of iPSC-derived MNs from control line OX3-9 with a short low molecular weight band and *C9orf72* patient iPSC-derived MNs (T2-6, T2-7, 7245-1 and 7245-3) with high molecular weight bands, indicative of repeat expansions. C) Absence of repeats was confirmed by repeat-primed PCR for the control line OX1-19, and repeats could be detected by repeat-primed PCR for C9-7245-3 MN line.

**
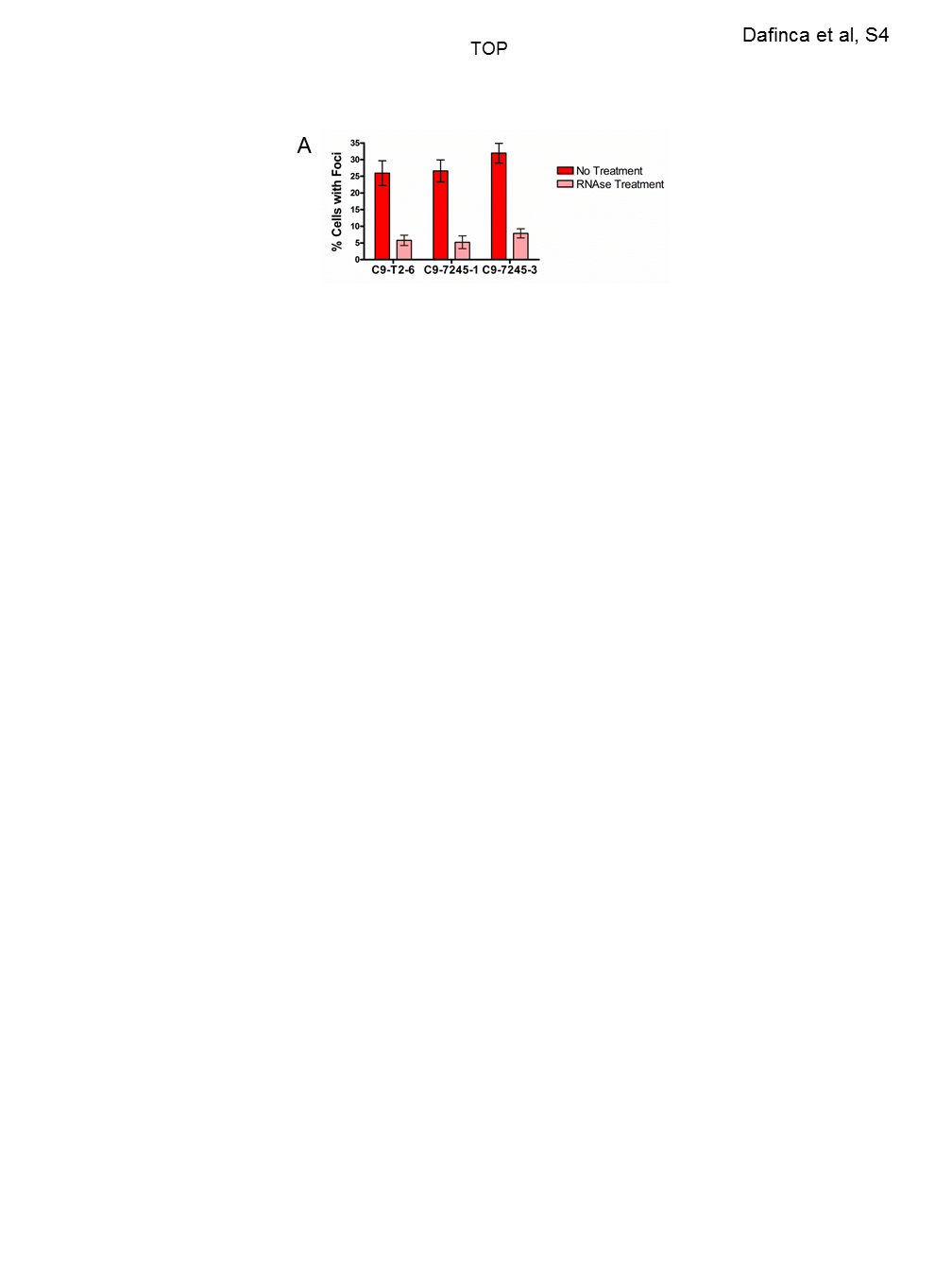
**

**Figure S4: Confirmation of specificity of the CCCCGG probe** A) Quantification of RNA foci with or without the addition of RNAse confirms the specificity of the probe. Data are represented as average±SEM.


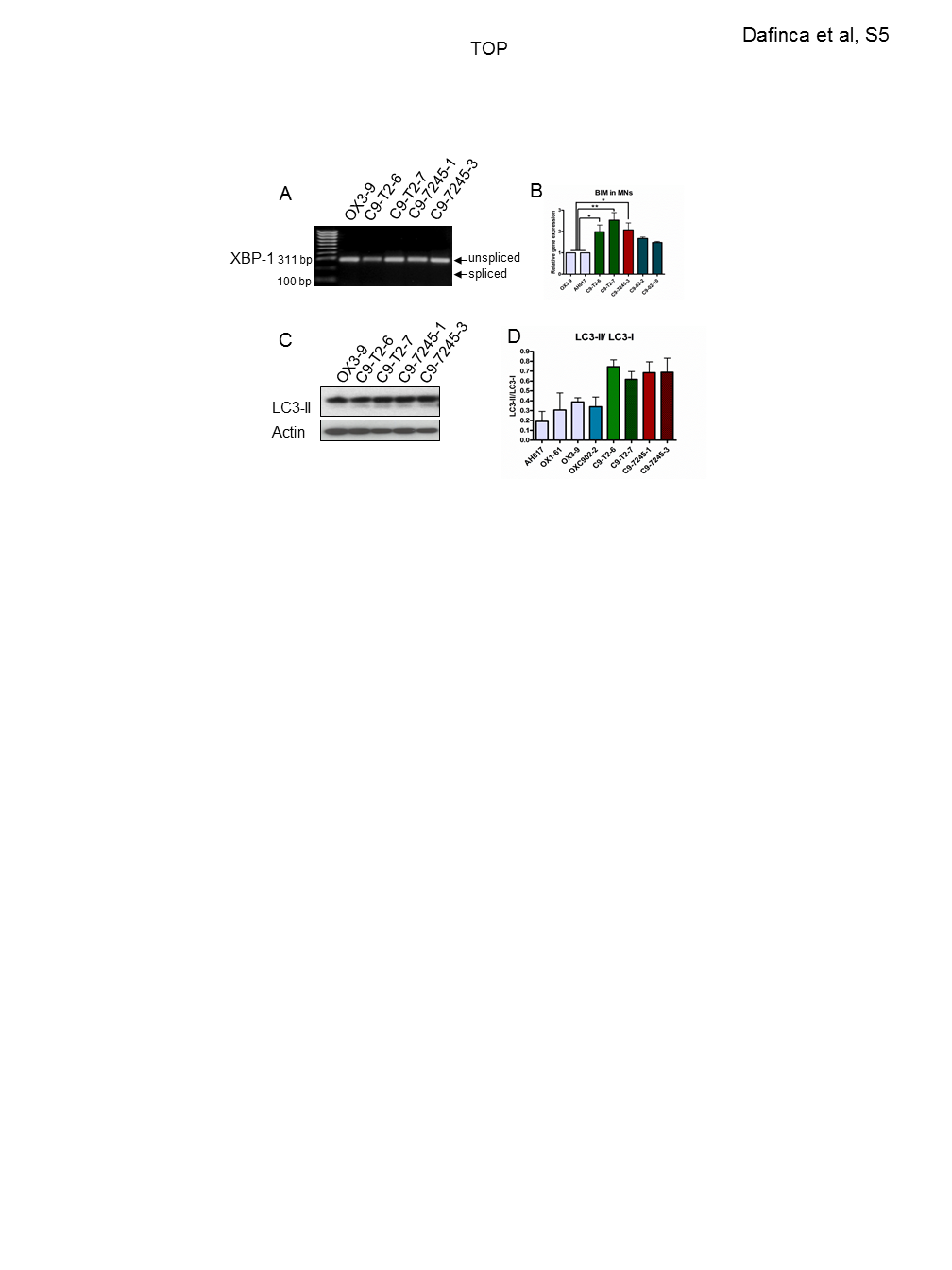


**Figure S5: Analysis of XBP-1, BIM and LC3-II.** A) No differences are detected in XBP-1 splicing. B) qPCR of the pro-apoptotic BIM shows significantly higher levels of expression in C9-T2 and C9-7245-1 compared to controls (**p*<0.05; ***p*<0.01, One-Way ANOVA with Dunnett’s *post hoc* test). C) Immunoblotting of LC3-II and D) quantification shows no significant difference in LC3-II expression between the controls and *C9orf72* MNs (One-Way ANOVA).
